# IRFinder-S: a comprehensive suite to discover and explore intron retention

**DOI:** 10.1186/s13059-021-02515-8

**Published:** 2021-11-08

**Authors:** Claudio Lorenzi, Sylvain Barriere, Katharina Arnold, Reini F. Luco, Andrew J. Oldfield, William Ritchie

**Affiliations:** Institut de Génétique Humaine, Centre National de la Recherche Scientifique (CNRS), Université de Montpellier, Montpellier, France

**Keywords:** Intron retention, Splicing efficiency, RNA sequencing

## Abstract

Accurate quantification and detection of intron retention levels require specialized software. Building on our previous software, we create a suite of tools called IRFinder-S, to analyze and explore intron retention events in multiple samples. Specifically, IRFinder-S allows a better identification of true intron retention events using a convolutional neural network, allows the sharing of intron retention results between labs, integrates a dynamic database to explore and contrast available samples, and provides a tested method to detect differential levels of intron retention.

**Supplementary Information:**

The online version contains supplementary material available at 10.1186/s13059-021-02515-8.

## Background

Intron retention (IR) occurs when an intron is transcribed into pre-mRNA and remains in the final mRNA. It is a type of alternative splicing that is gaining increased interest in human health and disease research. Originally described in plants and viruses, IR has now been shown to be a common form of alternative splicing in mammalian systems with a major impact on normal biology and disease [1–7]. However, detecting IR events poses several specific difficulties. Introns are highly heterogeneous genomic regions, both in length and sequence features. In mammals, IR levels are generally low and thereby subject to incomplete coverage and higher count overdispersion. As a result, software that is not specifically tuned for IR detection generally performs poorly and databases that provide transcript isoform sequences fail to list many IR events [4, 8].

We previously published a method called IRFinder, an algorithm for detecting and quantifying IR events, that is frequently used as a benchmark for IR detection and quantification [8–12]. This software and its associated database have been critical in the detection and interpretation of IR events in numerous studies [13–19]. However, building on 4 years of user feedback, it is apparent that IRFinder is lacking features that would enable bench scientists to more reliably identify actionable IR events, share IR data, and dynamically analyze changes in IR levels between multiple samples. We have implemented a suite of features in a new version of our software called IRFinder-S. Specifically, we have (1) created a dynamic database that allows users to perform a meta-analysis, contrast IR from multiple samples, and view IR in an internal browser; (2) created an infrastructure allowing users to share IR detection results from their own samples; (3) implemented a convolutional neural network that analyzes genomic coordinates, as a genome browser would display, and pinpoints IR events that are most likely candidates for further wet-lab analysis; (4) implemented IR detection from third-generation long sequencing technologies; and (5) implemented and tested differential analysis of IR levels between samples.

## Results and discussion

### IRBase enables the visualization and contrast of IR events as well as data sharing

**Fig. 1:**
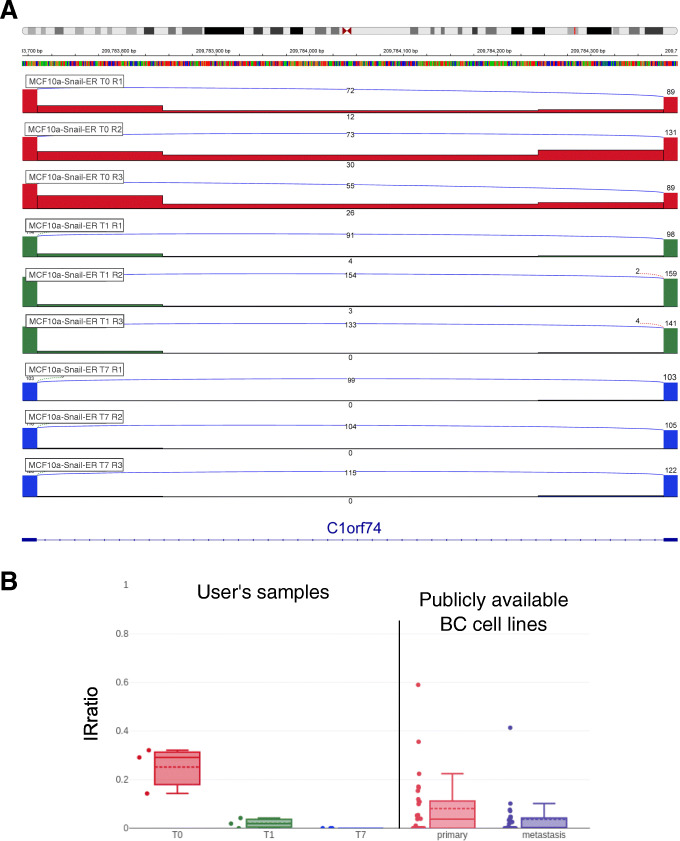
IRbase 2.0 is a web application that allows users to visualize and share IRFinder results. **A** IGV view of three replicates of MCF10a-Snail-ER cells without tamoxifen treatment (T0, in red), after 1 day of treatment (T1, in green) and after 7 days (T7, in blue) using the new IRFinder track type. The BED graph track style of the same locus is represented in Additional file 1: Fig. S1. **B** Boxplot showing the IRratio in the nine user provided samples and in 55 breast cancer cell lines, 29 primary tumors, and 26 metastasis, currently publicly available in IRbase

It is essential to visualize and contrast specific intron retention events detected by computational approaches before spending resources on their experimental validation. This allows users to understand the transcriptional context of a predicted IR event but also to assess whether the event is common to other cell types or specific to their experiment of interest. We therefore created a web application that allows users to upload their own data, decide whether to keep them private, or share them with other users and visualize the results in a javascript version of the IGV genome browser. We propose two types of tracks to visualize the IR events: a bar mode, showing the ratio values like a BedGraph and an IRFinder track to visualize the abundances of the flanking regions, the number of reads spliced and intron read depth (Fig. 1A and Additional file 1: Fig. S1). These views can integrate results from publicly available datasets and shared data from other users (Fig. 1B). Currently, IRbase accepts results from hg38 and ENSEMBL annotation and contains 935 cell lines (downloaded from https://portals.broadinstitute.org/ccle). This database is fully integrated within the IRFinder detection tool; users who have predicted IR events using our software are prompted to upload and share their results. By facilitating the upload process and allowing easy integration using flexible labelling of experiments using user-defined tags, we ensure that the database can grow steadily. The database is accessible for meta-analyses across tissue types and conditions and allows users to contrast multiple experiments in one interface.

### IRFinder-S integrates long read detection of IR

**Fig. 2:**
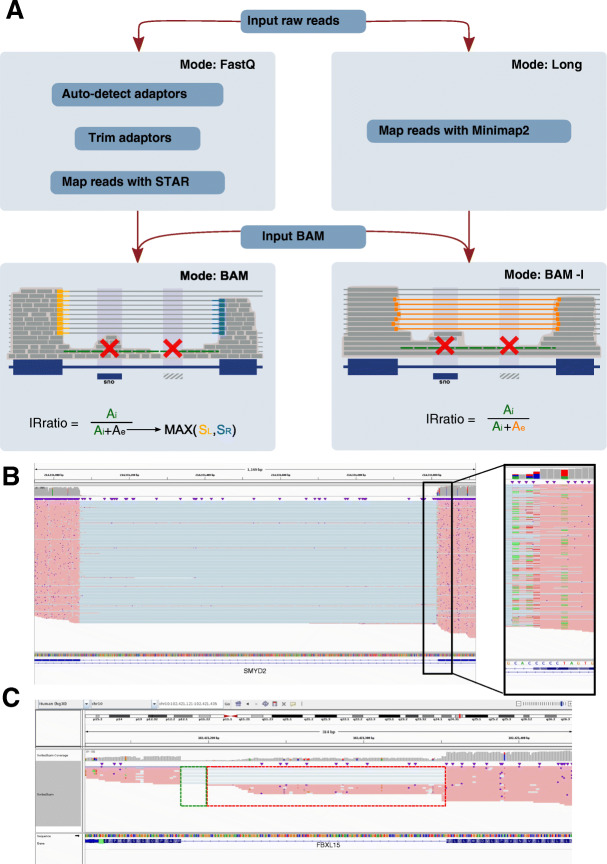
IRratio estimation for long reads. **A** Main changes in the pipeline between short reads (left) and long reads (right). *A*_*i*_ = intron abundance and *A*_*e*_ = exon abundance. In short reads, *A*_*i*_ is estimated as the median value of the intron depth; in long reads, we use the minimum intron depth. The exon abundance in short reads is estimated as the maximum value between the number of reads spliced at the 5′ or 3′ of the intron; in long reads, it is the number of reads that are spliced in 5′ and 3′. Red crosses indicate regions that will be excluded from further analysis either due to other overlapping transcripts (left cross) or low mappability regions (right cross). Green lines indicate intronic abundance (*A*_*i*_). **B** Example of poor alignment to exonic borders due to sequencing errors that creates a jitter effect on the mapping of long reads around the splice junctions. Without jitter (option -j 0), the software identifies only 110 SpliceLeft reads, 0 SpliceRight, and 0 ExactSplice. With the jitter option (-j 3), it identifies 183 SpliceLeft reads, 210 SpliceRight reads, and 69 ExactSplice. Having an intron abundance (*Ai*) of 4 and using the ExactSplice as Ae, the IRratio in the first case is 1 and it raise the LowCover warning; in the second case, the IRratio is 0.055, raising the MinorIsoform warning due to the imbalance between the ExactSplice and the max(SpliceLeft, SpliceRight)”. **C** Example of alternative 3′ end that is considered as intron retention by the standard method (IRratio=0.40, due to the reads in the red box that do not extend fully between the two exons) and not by the long read mode (IRratio=0, due to the absence of reads covering the green and red boxes)

Third-generation sequencing technologies, especially direct RNA sequencing, represent a unique opportunity for the detection, characterization, and validation of IR. Because these technologies are capable of sequencing individual RNA molecules from start to end, they can elucidate the full structure of transcripts with retained introns. As a consequence, long reads can be considered as a means of validating IR predictions obtained from SR data. The increased availability of long reads facilitates the study of splicing structure, including a more reliable identification of IR events. IRFinder-S proposes a dedicated version of the algorithm for long-read sequencing (Fig. 2 and Materials and Methods). In this long-read mode, we make multiple adjustments to the algorithm to account for the specificities of long-read data but also to account for the fact that these reads will often serve as the validation of IR and thus the default parameters are more stringent. Firstly, the mapping algorithm STAR is replaced by Minimap2 [20], a specialized aligner for long reads providing competitive alignment accuracy and low computational requirements. Secondly, because long-reads have a higher error rate that often leads to slight imprecision in the definition of exonic boundaries (Fig. 2B), we allow by default up to three nucleotide jitter in exonic boundaries when calculating correctly spliced introns (parameter -j). Thirdly, we only consider the minimum read depth rather than the median when considering retained intron abundance. These modifications allow us to use more long reads when measuring IR levels and also filter out reads for which IR calls would be uncertain (Fig. 2C).

### Convolutional neural networks enable users to pinpoint actionable IR events

**Fig. 3:**
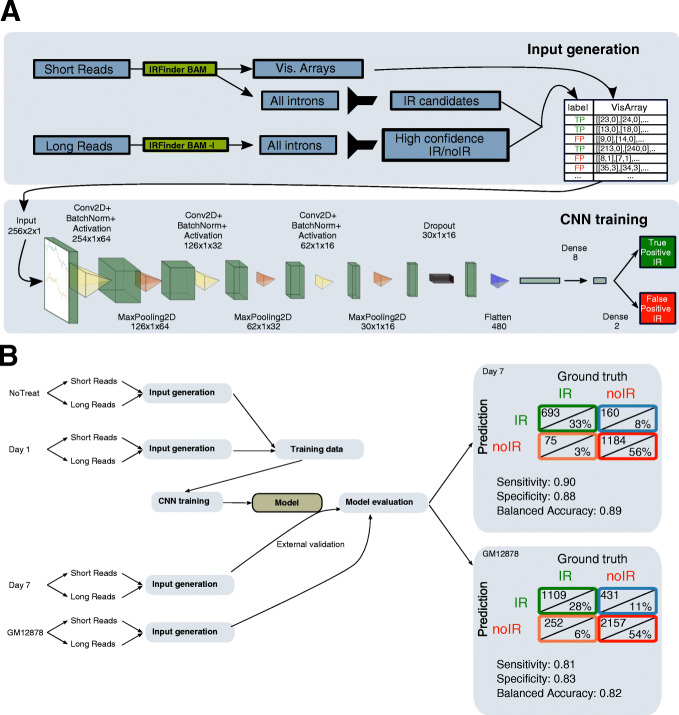
Convolutional neural network (CNN) filter to reduce false-positive IR candidates. **A** Top: Workflow for the generation of the training input: the short reads are used to generate the training array and to determine if, based uniquely on the IRFinder output, each intron would be considered as retained or not. The long reads are used to determine the IR ground truth. Bottom: the architecture of the CNN model. **B** Training and evaluation of the model using the external datasets; the ground truth is determined using the presence or absence of coverage in the long reads

Feedback from the users of our first version of IRFinder confirmed that visual inspection of IR events was a crucial step in selecting candidates. Specific patterns that an expert could detect in a genome browser increased the likelihood of selecting good candidates. Features such as the regularity of intronic coverage, the presence of well-defined exons, and other features contributed to the review of IRFinder candidates. However, this process is time-consuming and variable from user to user. Thus, we tried to reproduce this expert viewing by using a deep-learning approach that would detect these patterns from a dataset of high-quality IR events. To this end, we trained a convolutional neural network (CNN) using high confidence retained introns confirmed by long reads as ground truth. This CNN filter is directly integrated into IRFinder, and it works by transforming coverage data into visual arrays that are submitted to the CNN (Fig. 3A). To test this approach, we used an inducible cell reprogramming system based on human MCF10A cells that recapitulates the epithelial-mesenchymal transition (EMT, Materials and Methods) for which we had access to both short- and long-read RNA-seq data (Fig. 3B). In this system, MCF10a cells stably express the EMT-inducing transcription factor Snail fused to the estrogen receptor. Upon treatment with tamoxifen, the first changes in alternative splicing can be observed as soon as 24h, while a complete cell reprogramming is reached upon 7 days of treatment. We thus used as a training set three biological replicates of untreated epithelial cells and three replicates treated for 1 day with tamoxifen, which corresponds to the first day of the EMT transition. As a first external validation set, we used three biological replicates of cells treated for 7 days with tamoxifen, corresponding to the fully induced mesenchymal-like state. This division aims to validate the model on new IR events that are likely to emerge in the mesenchymal-like state and therefore never seen by the model in the training dataset. As a second external validation set, we used long-read data of GM12878 B-Lymphocite cell lines, provided by the nanopore consortium [21]. Because there was no short read (SR) dataset provided with this experiment, we used the GM12878 Illumina data from an earlier ENCODE study, processing the data as described in our previous study [22]. We considered IR events detected in both short reads and long-reads as bonafide IR events to measure true positives (Material and Methods). We trained the model to recognize the true positive introns from the false positive ones in a 10-fold cross-validation procedure. We then evaluated our model on a biologically distinct dataset where the cells had fully transitioned to their mesenchymal-like state. On this independent test set, it achieved a sensitivity of 0.90 and a specificity of 0.88, with a balanced accuracy of 0.89 (Fig. 3B, right). We then evaluated our model on a different cell line, GM12878, where the model achieved a sensitivity of 0.81, specificity of 0.83, and a balanced accuracy of 0.82.

We then benchmarked IRFinder-S against iREAD [12], a recent software dedicated to the analysis of intron retention, MAJIQ [23], a software designed for the analysis of alternative splicing events that adjust the PSI value of retained intron, and Whippet [24], another software that uses fastq files to compute PSI values. These software were selected based on their popularity but also on whether they could output a measure of retained versus spliced out introns. The results are shown in Table 1 and Additional file 1: Fig. S2. It is worth noting that Whippet excludes a high number of introns prior to their quantification steps since it builds its reference based only on known retained introns and would thus be unable to detect rare or unannotated IR events. iREAD excludes all the introns overlapping with other features. For IRFinder, we excluded the introns reporting any warnings. IRFinder-S achieves the best overall performance, excludes the least introns before analysis, and thanks to the CNN it does not require the user to set a threshold on IR ratio. To benchmark execution time, we ran a single sample (the third replicate of the EM test sample) on a single core. IRFinder-S processed a single BAM in 20 min, MAJIQ 31 min, and iREAD 50. Whippet took 194 min to process a sample; however, Whippet starts from FASTQ files instead of already aligned BAM files, for which the alignment takes 120 min using STAR on a single core. Interestingly, when we add the CNN on top of the other benchmarked algorithms, it reduces the number of false positive introns, at the expense of a small number of true positives (Additional file 1: Fig. S3) making the CNN a valuable approach for our algorithm but also for other approaches. An example of an intron correctly filtered out by the CNN is presented in Additional file 1: Fig. S4.

**Table 1:** Table representing the results of the benchmark on the EMT test dataset (**A**) and on the GM12878 test dataset (**B**) using a threshold for the PSI values and IR ratios of 0.10

Method	Excl.	TP	TN	FP	FN	TPR	TNR	PPV	Acc.	FDR
**A.** EMT test results										
IRFinder	**25989**	695	95198	428	138	**0.83**	0.99	0.62	0.98	0.38
IRFinder-S	**25989**	673	35515	111	160	0.81	**1.00**	**0.86**	**0.99**	**0.14**
iREAD	28221	18	33994	86	129	0.12	**1.00**	0.17	**0.99**	0.83
Whippet	59822	443	1978	87	118	0.79	0.96	0.84	0.92	0.16
MAJIQ	30179	388	29572	1951	358	0.52	0.94	0.17	0.93	0.83
**B.** GM12878 test results										
IRFinder	**30943**	1228	50720	729	185	0.87	0.99	0.63	0.98	0.37
IRFinder-S	**30943**	1077	51123	326	336	0.76	0.99	**0.77**	**0.99**	**0.23**
iREAD	37905	71	45501	179	149	0.32	**1.00**	0.28	**0.99**	0.72
Whippet	80125	772	2459	347	102	**0.88**	0.88	0.69	0.88	0.31
MAJIQ	50932	826	30626	917	504	0.62	0.97	0.47	0.96	0.53

*Excl* intron excluded, *TP* true positive, *TN* true negative, *FP* false positive, *FN* false negative, *TPR* true positive rate (sensitivity), *TNR* true negative rate (specificity), *PPV* positive predicted value (precision), *Acc.* accuracy, *FDR* false discovery rate

Inspection of examples where the CNN was mistaken reveal that the same mistakes would probably have been made by visual examination by an expert; the false positives generally present a homogenous coverage across the intron (Additional file 1: Fig. S5A top right) and false negatives seem to present unevenly covered intronic regions (Additional file 1: Fig. S5A bottom left). Finally, the performance of our CNN may be underestimated because the misclassified IR events are generally borderline with IRratios close to the threshold of 0.1, and mislabeled introns, due to incongruences between long- and short-read resolution (Additional file 1: Fig. S5B).

### Implementation and validation of differential IR analysis

In our first version of IRFinder, we suggested methods to analyze differential IR (DIR) using either standalone scripts written in a different coding language or a procedure requiring the user to have extensive knowledge of data transformation and statistical languages such as R. In IRFinder-S, we include IRFinder Diff, an integrated method that allows end to end analysis using either the density-based approach, DESeq2 [25], or the PSI-based approach, SUPPA2 [26] adapted for IR ratios (Material and methods). The output can be used in SUPPA2 downstream analysis for clustering analysis for example. Our choice of algorithms was based on the popularity of these two approaches for the analysis of transcriptomic data. We now wanted to test if they were suitable for the detection of differential IR.

**Fig. 4:**
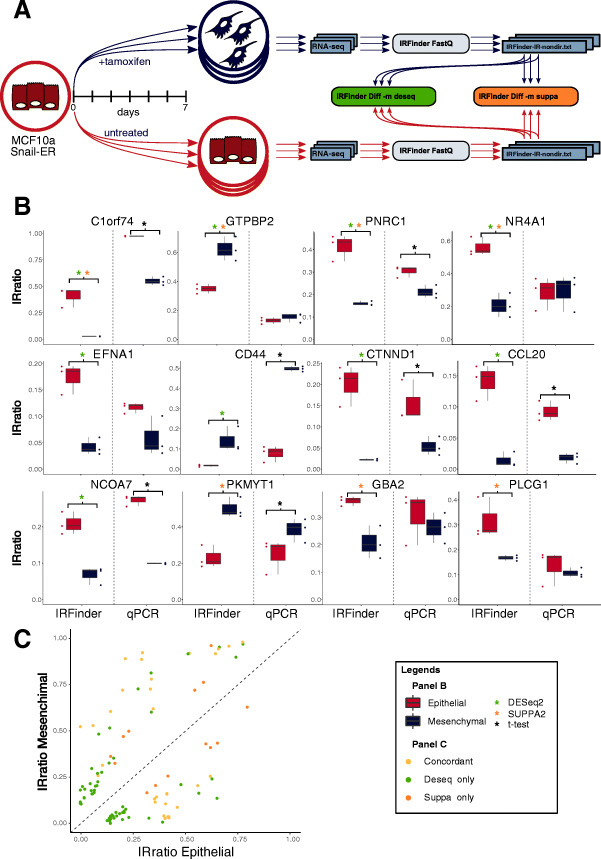
Differential Intron retention validation between stages of EMT differentiation. **A** EMT is induced in MCF10a Snail-ER system. RNA-seq data is analyzed with IRFinder, and the replicates are compared using DESeq2 and SUPPA2 wrappers. **B** qPCR validation of 12 introns called as differentially retained by DESeq2 and/or SUPPA2. The complete list of primers and characteristics of each intron is described in supplementary file 1. **C** Scatter plot showing the IRratios of the introns called as differentially retained by DESeq2, in green, SUPPA2, in orange, and by both in yellow

In order to corroborate and compare DESeq2 and SUPPA2 as methods to identify differentially retained introns, we used the aforementioned EMT system (Materials and methods). We compared three replicates of EMT-induced MCF10a cells (mesenchymal-like state) and three untreated control replicates (epithelial state) to detect differentially retained introns between the mesenchymal and epithelial states (Fig. 4A). Using standard settings for both algorithms (BH adjusted *p* value < 0.05 for both, absolute FC > 1.5 for DESeq2 and delta ratio ≥ 0.1 for SUPPA2), we found that DESeq2 identified 148 differentially retained introns and SUPPA2 found 46 (Additional file 2: Table S1 and Additional file 3: Table S2). 31 differential IR events were common between the two. In both cases, introns were considered if at least one sample had IRratio > 0.05.

We selected 12 introns called as differentially retained and that were suitable for clean primer design in that they did not overlap with other exons or have any known alternative donor or acceptor sites. The selected introns were the following: four introns, in the genes C1ORF74, GTPBP2, PNRC1, and NR4A1 called by both methods; six introns, in the genes EFNA1, CD44, CTNND1, CLL20, and NCOA7, called only by DEseq2; and 3 introns, in the genes PKMYT1, GBA2, and PLCG1, called only by SUPPA2. Figure 4B shows the delta IRratios between epithelial and mesenchymal replicates as computed by IRFinder and the ones obtained by qPCR validations. Of the 12 tested introns, 7 were confirmed using RT-qPCR. The comparison between IRFinder-S and RT-qPCR results showed that both approaches display comparable changes between epithelial and mesenchymal IR ratios. However, we observed that DESeq2 identifies more DIR events in samples with an average lower IRratio (Fig. 4C). This may be explained by the fact that events with low intronic coverage produce highly variable IR ratio values. As a consequence the ratio values may be highly variable within replicates and methods such as SUPPA2 which make use of replicate variability to determine uncertainty may not produce statistically significant scores. As such, DESeq2 is chosen as the default with SUPPA2 available if required.

## Conclusion

Until recently, IR detection ran parallel with the analysis of other splicing events without taking into account inherent difficulties in measuring intronic expression. As a result, IR has been systematically underestimated. Despite the recent development of specialized software for detecting IR, the measurement of IR levels has been problematic. Here, we introduce IRFinder-S to overcome major obstacles in IR detection and exploration. These include a database to explore IR in numerous tissue types and share IRFinder results, the addition of a CNN filter to drastically reduce the false-positive rate of IR detection, the inclusion of an experimentally validated approach to detect differential IR, and the ability to analyze long-read sequencing data. In addition, IRFinder-S overcomes many issues unveiled in the last 4 years thanks to community feedback, such as the possibility to give pre-computed low mappability areas, whose creation step takes most of the time during the reference creation, the possibility to link pre-existing STAR reference folders, and a detailed help divided by run modes. Finally, Docker and Singularity images including all the dependencies required to run IRFinder on any Linux distribution are available in dockerhub (cloxd/irfinder:2.0) and in GitHub (https://github.com/RitchieLabIGH/IRFinder).

## Methods

### IRbase 2.0

The new version of IRbase consists in a frontend, implemented with Angular 10, a mySQL database containing the basic information about each sample submitted and the introns having IRratio higher than 0.05, warning different than “LowCover” and a tag-based aggregation system that allows fast queries to obtain statistics on large number of samples.

The backend is implemented in node express version 4.17.1. We generated two novel tracks to show IRFinder results (IRFinder-IR-[non]dir.txt files) directly on igv.js, one displaying the IRratio as bedgraph and one that combines the additional information included in the file allowing the representation in detail of the flanking exons, the spliced reads, and the intron depths, as shown in Fig. 1.

The user authentication is managed by Google’s service firebase and is necessary in order to upload new samples. Currently, IRbase requires results from hg38 with ENSEMBL reference.

### Measuring intron retention in long reads

In order to adapt the IRratio computation in long read, we adapted the estimation of intron and exon abundance keeping unchanged the formula:

$$ IRratio=\frac{Intronic\ abundance}{\left(\  Intronic\ abundance+ exonic\ abundance\ \right)} $$

A visual representation of the main changes is shown in Fig. 2. The intron abundance in long reads is evaluated as the minimum coverage in the intron instead of the median, offering a more stringent but reliable IRratio. The exon abundance in long reads is estimated as the exact number of reads spliced between the acceptor and the donor site, rather than the highest number of reads spliced between donor and acceptor sites. Finally, in order to take into account the long reads’ higher error rate, the count of the splits is considered not only for the exact split nucleotide annotated but also the three flanking positions.

This alternative version is used by default in IRFinder long mode and is triggerable by the “-l” flag argument using IRFinder BAM.

### Convolutional neural networks

The network was trained on the epithelial datasets labeled T0 and T1 (days 0 and 1 of treatment) and validated on the mesenchymal dataset T7, described in our previous work [22] and having biological samples sequenced with both unstranded short and stranded long-read technologies. We use IRFinder to analyze the raw data, and for each pair of data belonging to a sample, we selected the introns with IRratio above 0.05 and no warnings in short reads, as putative IR candidates. We then used the long reads as ground truth of the corresponding intron: we labeled as true positive IR, the introns with no warning, depth (intron abundance + exon abundance) of 25, and IRratio above 0.1 and as false positive IR, the introns with 50 depth and IRratio of 0. Our rationale is that it is easier to assert the existence of IR events than to assert their absence; thus, we pushed the required depth for negative events to 50 to increase their likelihood of being true negatives.

To allow the model to use directional and non-directional libraries and to reduce mislabeled events, we considered only the introns having a congruent label between the directional and non-directional long reads IRFinder results. Due to the scarcity of FP, we included in the training set also true negative introns having IRratio higher than 0.01 in the SR to ensure a balanced dataset.

### Benchmark

To compare IRFinder’s results with the output of iREAD [12], Whippet [24] (v1.6.1) and MAJIQ [23] (Build v2.1-c3da3ce), we used the reference genome hg38 and ENSEMBL v100 annotation, generating the required reference files for each software. We paired the results of each method with the introns of the ground truth determined from the long reads in the test datasets as described in the previous chapter.

We used two arbitrary thresholds, 0.05 and 0.10, for the PSI values of Whippet and IRFinder’s IRratio to classify the introns in IR and non-IR. For what concerns MAJIQ, we considered as no IR the introns without a PSI value adjusted for intron retention and the introns having an adjusted PSI value lower than the two arbitrary thresholds.

### Differential intron retention

The DESeq2 constructor is used to fit a GLM based on the intronic abundance (intron depth column) and the exonic abundance (the maximum between LeftSplice and RightSplice) to test the fold change of IR between two conditions.

The SUPPA2 wrapper uses IRratio values instead of percent splice in (PSI) values, both spanning from 0 to 1, and the exon abundance instead of transcript per million (TPM) values, considering so far the expression of the exons surrounding each intron rather than the average transcript expression.

In both cases, the user can decide to remove introns with warnings (by default, introns with LowCover in at least one sample are removed) and to set a threshold on the minimum IRratio that at least one sample has to meet (by default 0.05).

The command line interface offers a simple tool to use DESeq2 or SUPPA2 on two or more sets of samples, requiring only the location of the IRFinder result files IRFinder-IR-[non]dir.txt. In case of more than two sets, all the pairwise comparisons are reported in the output folder.

### Cell line culture

Non-transformed human female breast epithelial cells (MCF10a cells) were cultured at 37°C and 5% CO_2_ in DMEM/F12 (Sigma) supplemented with 5% horse serum (ThermoFisher), 10 ng/ml EGF (Sigma), 10 μg/ml insulin (Sigma), 0.1 μg/ml cholera toxin (Sigma), 0.5 μg/ml hydrocortisone (Sigma), 1% l-glutamine (Sigma), and 1% penicillin/streptomycin (Sigma; culture medium). Cells were kept in high confluency (approx. 70%) in order to maintain their epithelial character and passed every 2–3 days by trypsinization (0.25% Trypsin (Sigma) for 15–20 min).

### Epithelial-mesenchymal transition (EMT)

MCF10a-Snail-ER cells were used as cellular model for EMT. In this model, EMT is induced by addition of exogenous 4-hydroxy-tamoxifen to the cells, which changes Snail-ER conformation and can thus be translocated to the nucleus for silencing of key epithelial markers and expression of mesenchymal genes within 24 h. Prior to induction, 850,000 cells were seeded in 15-cm culture plates and grown in 17-ml culture medium for approximately 24 h. Twelve hours before tamoxifen treatment, the cells were synchronized by exchanging the medium to serum free medium (culture medium without horse serum). Cells were incubated for 6 days in culture medium with 100 nM 4-hydroxy-tamoxifen (Sigma). Controls were performed by adding equivalent volumes of methanol.

### Primer design

We selected IR events by visual inspection, selecting introns without neither antisense transcript nor known exon in each sample and without excessive noise in the intron body. Two sets of primers were designed for each intron, one pair overlapping the exon-exon junction and one covering the intron-exon junction.

### RT-qPCR

RT-qPCRs were performed in biological triplicates. RNA was extracted from cells using QIAshredder (Qiagen, 79656) and GeneJET RNA purification kit (Thermo Scientific, #K0732) following the manufacturers’ instructions. 500 ng of the total RNA was DNase treated (Promega, M6101) and reverse-transcribed using oligo(dT) primers (Transcriptor First Strand cDNA Synthesis kit, Roche 04897030001).

For each biological replicate, qPCRs were performed in technical duplicates using Bio-Rad CFX-96 Real-Time PCR System and iTaq Universal SYBR green Super-mix (Bio-Rad #1725121). For each intron of interest, two primer pairs were designed that includes the exon-exon (of the flanking exons) and an intron-exon junction, respectively.

## Supplementary Information

**Additional file 1.** Supplemental Figures.

**Additional file 2.** Supplemental Table S1.

**Additional file 3.** Supplemental Table S2.

**Additional file 4.** Review history.

## Data Availability

Cell line data used to help populate the database was taken from: https://portal.gdc.cancer.gov. Direct RNA Nanopore and Illumina RNA-seq MCF10A samples have been deposited on GEO under accession number GSE126638 [27]. GM12878 cell line, the long read data, was available from the Nanopore consortium at https://github.com/nanopore-wgs-consortium/NA12878. We made use of the *Run1* (MinION ONT direct-RNA, kit SQK-RNA001, pore R9.4) generated by the UCSC laboratory. These long reads were corrected using short read data from the same cell line sequenced by a separate consortium. These data were available from the GEO website (https://www.ncbi.nlm.nih.gov/sra/SRX159827). After the quality control using FastQC, we kept and pooled together runs SRR521447, SRR521448, SRR521453, SRR521454, and SRR521455. An OceanCode capsule is available at https://codeocean.com/capsule/0822057/tree [28] that reproduces the main functionalities of IRFinder-S. IRFinder-S is available at https://github.com/RitchieLabIGH/IRFinder [29] under the MIT license.
