# Supplementary material for: IRFinder-S: a comprehensive suite to discover and explore intron retention
Source: Genome Biol. 2021 Nov 8;22:307. doi: 10.1186/s13059-021-02515-8 (PMC8573998; doi:10.1186/s13059-021-02515-8)
Supplement: Supplementary file 1 — Additional file 1. Supplemental Figures. [file 13059_2021_2515_MOESM1_ESM.docx]

Supplementary of IRFinder-S: a comprehensive suite to discover and explore intron retention

Claudio Lorenzi^1*^, Sylvain Barriere^1*^, Katharina Arnold^1^, Reini F. Luco^1^, Andrew J. Oldfield^1^, William Ritchie^1†^

1 Institut de Génétique Humaine, Centre National de la Recherche Scientifique (CNRS), Université de Montpellier, Montpellier, France

* These authors contributed equally to this work
†To whom correspondence should be addressed. Tel:+33 4 34 35 92 40

Email: william.ritchie@igh.cnrs.fr


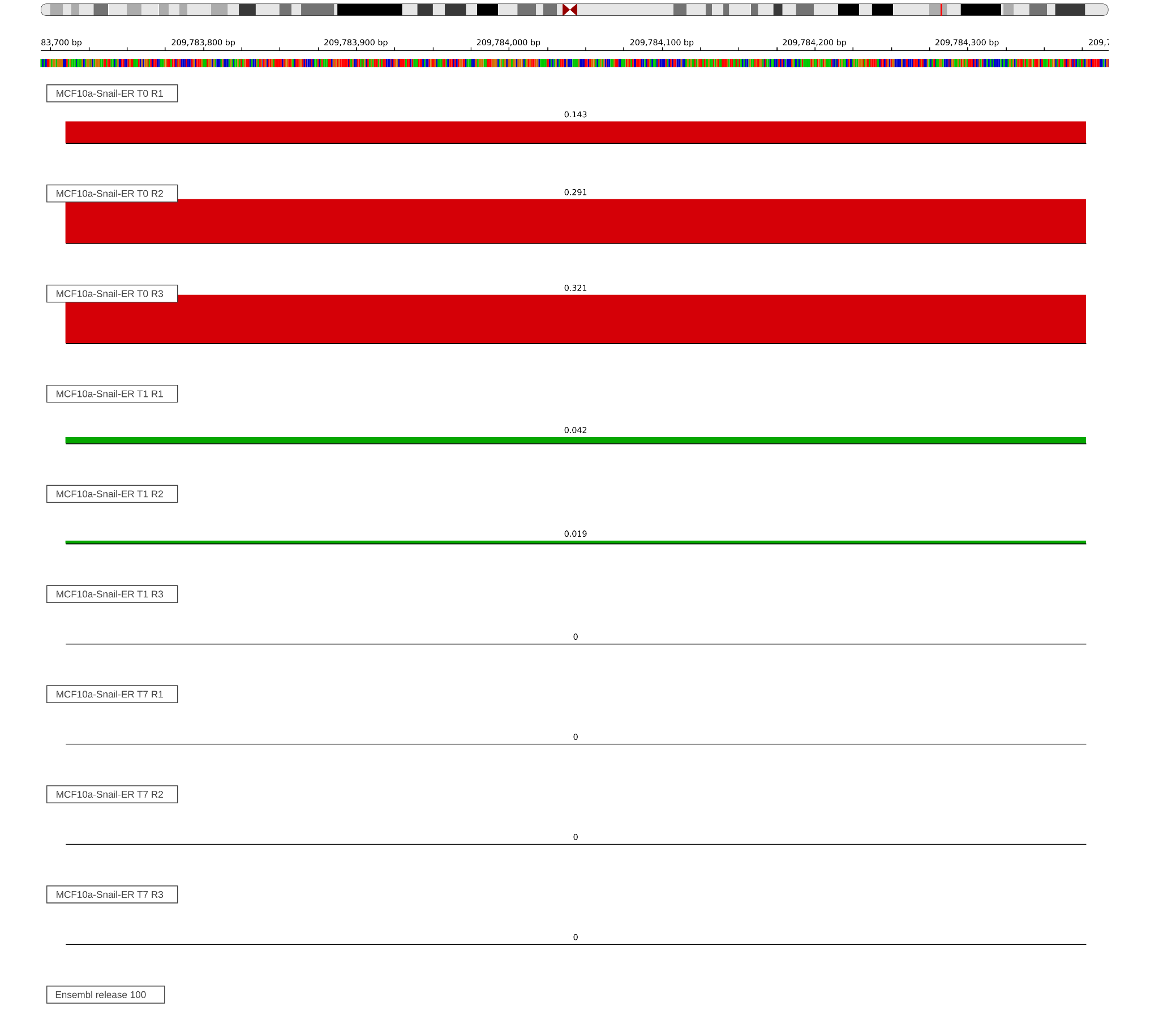


Figure S1: A) IGV view of three replicates of MCF10a-Snail-ER cells without tamoxifen treatment ( T0, in red), after one day of treatment ( T1, in green ) and after seven days ( T7, in blue ) using the BED graph representation.


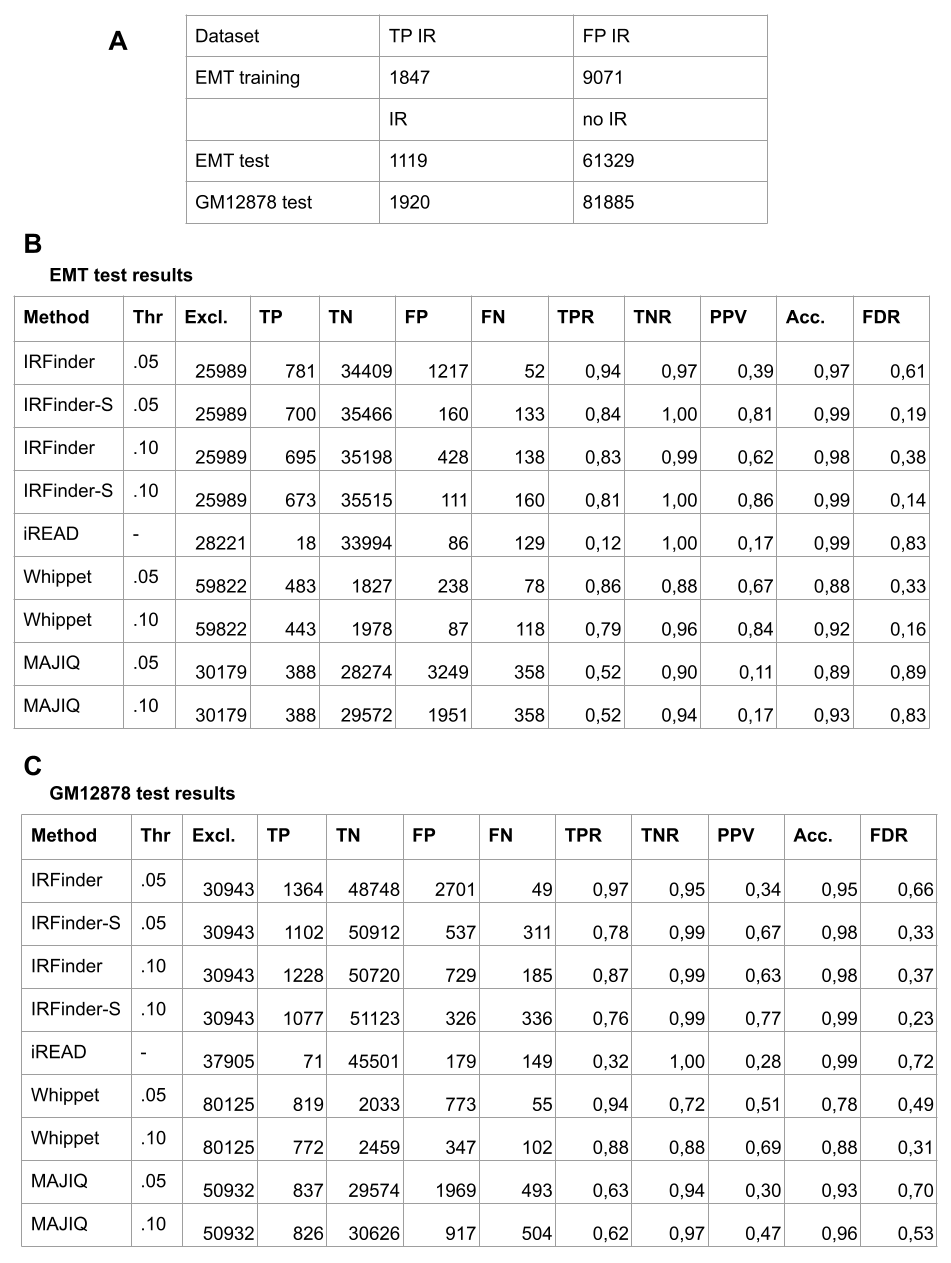


Figure S2: A) Number of introns used to train the model, retained introns and misclassified introns, and to test the softwares in the benchmark, retained introns and non retained introns, determined as described in Methods. B) Table representing the results of the benchmark on the EMT test dataset. C) Table representing the results of the benchmark on the GM12878 test dataset. Thr. = Threshold ; Excl = Intron excluded; TP = True Positive; TN = True Negative; FP = False Positive; FN = False Negative; TPR = True Positive Rate ( Sensitivity); TNR = True Negative Rate ( Specificity); PPV = Positive Predicted Value ( Precision); Acc. = Accuracy; FDR = False Discovery Rate.


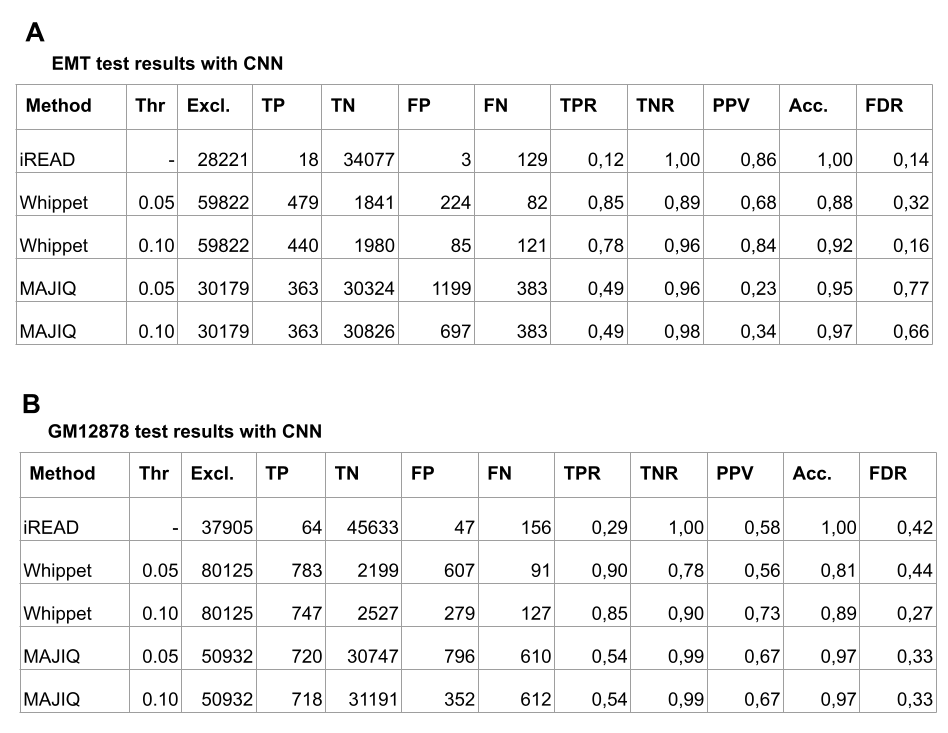


Figure S3: A) Table representing the results of the benchmark on the EMT test dataset adding the CNN filter to each method. C) Table representing the results of the benchmark on the GM12878 test dataset adding the CNN filter to each method.


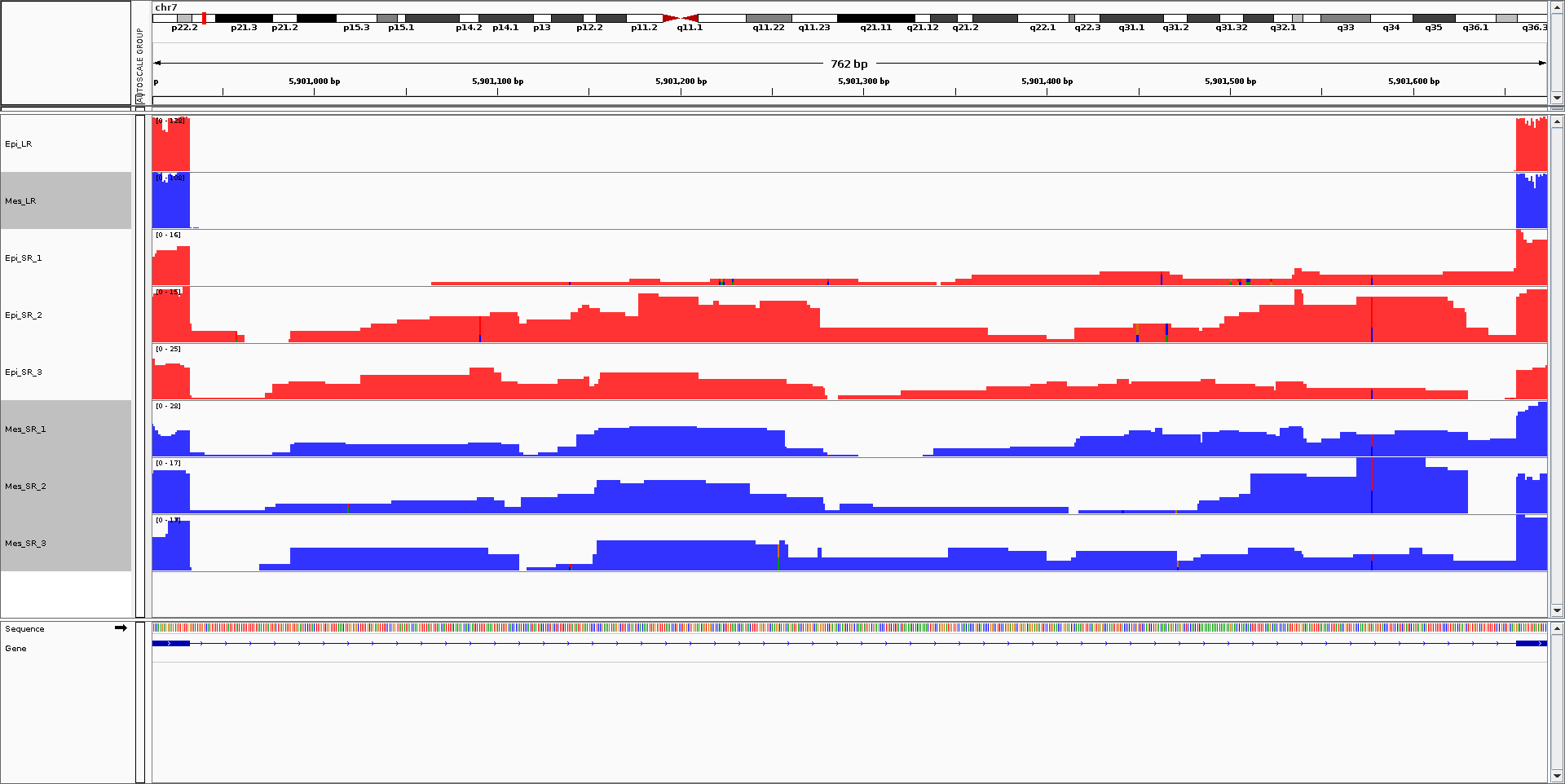


Figure S4: IGV screenshot of a false positive IR discarded by the CNN filter. The samples here represented are of the MCF10a cell line, treated with tamoxifen to induce the epithelial state ( blue tracks ) and untreated ( red tracks). The first two tracks represent the long reads where none of the mapping reads span across the whole intron. The following tracks show the alignment of the short reads, where some reads map in the intronic region, generating IRratios of respectively 0.15, 0.41, 0.32, 0.40, 0.29 and 0.25. All the SR would have been selected as IR candidates with the exception of the second Mesenchymal replicate ( track number 7 ), being the only one having a warning message ( NonUniformIntronCover ).


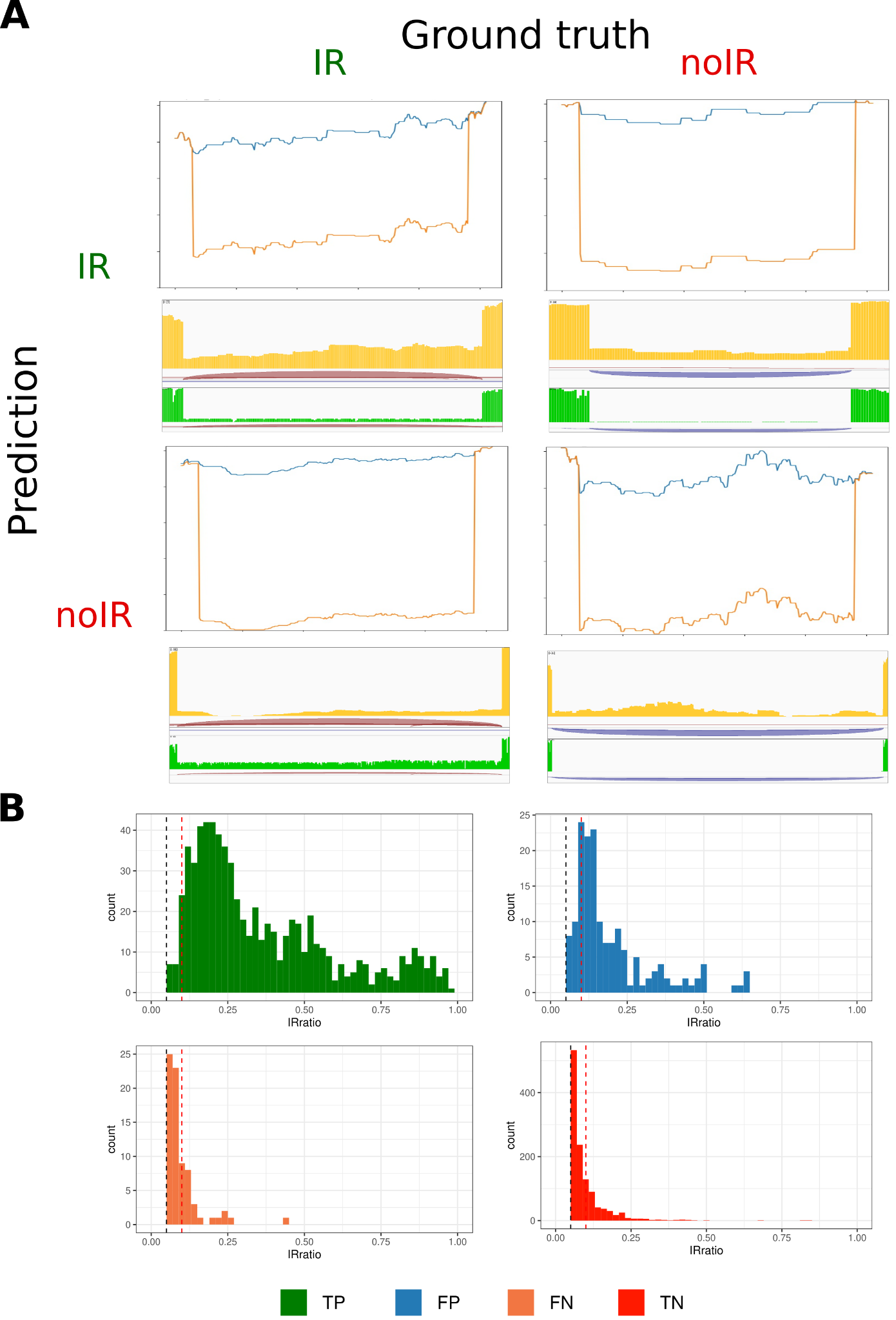


Figure S5: A) Four examples of events predicted by the CNN model compared with the ground truth determined by long reads. For each cell of the matrix: the CNN input on the top (orange lines represent the coverage, blue line the number of reads that are split across splicing junctions), the IGV view of the short reads in the middle in orange with red and blue arcs representing the junctions and the IGV view of the long reads on the bottom in green. B) Distribution of the IRratio in the analysed samples across the four classes of the confusion matrix. Most of the mislabelled events (FN and FP) have low IRratio. FP=False Positive, FN=False Negative, TP=True Positive, TN=True Negative
