# Supplementary material for: IRFinder-S: a comprehensive suite to discover and explore intron retention
Source: Genome Biol. 2021 Nov 8;22:307. doi: 10.1186/s13059-021-02515-8 (PMC8573998; doi:10.1186/s13059-021-02515-8)
Supplement: Supplementary file 4 — Additional file 4. Review history. [file 13059_2021_2515_MOESM4_ESM.docx]

Review History

**First round of review**

**Reviewer 1**

**Were you able to assess all statistics in the manuscript, including the appropriateness of statistical tests used?**

Yes, and I have assessed the statistics in my report.

**Were you able to directly test the methods?**

Yes

**Comments to author:**

The authors present a new suite of tools, IRFinder-S, for the detection and exploration of intron retention (IR) events from both short- and long-read sequencing of multiple samples, as well as for the detection of differential levels of IR between groups of samples. IRFinder-S builds on their previous software IRFinder and its highlighted novel feature is a convolutional neural network to reproduce expert identification of true IR events.

This manuscript is conceptually sound and IRFinder-S has the merit of implementing solutions for sharing and visualisation of data, levering the benefit of using long-read sequencing in the detection of IR events, and exploring the potential of deep learning in systematically reproducing expert examination of results. However, although we find no reasons to doubt that IRFindeR-S can satisfactorily (by current standards) quantify IR events, there are some shortcomings in the suite's benchmarking, detailed below, that need to be addressed for the edge brought by IRFindeR-S on the current state-of-the-art to be convincingly demonstrated. Moreover, we identified several needs of clarification, also listed below, that we encourage the authors to address.

Major concerns:

1. The deep learning model implemented to predict true, "actionable" IR events validated in a very specific biological context, as the training and testing steps were performed considering the EMT in the same cell line (MCF10A). Although there certainly are changes in gene expression and IR in that transition, the model is trained and tested on a limited set of events and therefore its evaluation is performed in a specific biological setting and not compared with any other scenario. For instance, would the predictive power of the model be similar if a different cell line was considered for external validation?

2. Along the same lines, the CNN filter to reduce false-positive intron retention events is applied only on IRFinder results. How does the described performance compare to that of applying the CNN to the calls of other computational tools available to perform IR detection?

Other essential revisions:

3. The figure legends and captions need serious improvements, as they lack important clarifying details. Regarding Figure 1A, in the main text the authors propose "two types of tracks to visualize the IR events: a bar mode, showing the ratio values like a BedGraph and an IRFinder track to visualize the abundances of the flanking regions, the number of reads spliced and intron read depth" but the different visualization modes are not made explicit in its legend or caption.

4. Similarly, from the caption of Figure 2B alone, the reader is not able to grasp what sort of "sequencing errors" are depicted. There is a zoom-in effect represented but left undetailed on what it represents.

5. In Figure 2A, the meaning and interpretation of the red crosses and the green lines should be made explicit in the caption or the figure itself.

6. In Figure 2C, the caption sentence "due to the absence of reads covering the green box" is misleading, as long read mode will require reads that span across the full intron, i.e., both red and green boxes. The current version suggests that reads covering only the green box are be considered.

7. In Results and Discussion' section "IRBase enables the visualization and contrast of IR events as well as data sharing", when referring to the improved upload process provided in the presented database, it is not clear what "fast labelling of experiments" actually means, making it difficult to assess how faster this is compared to other approaches. How long does this task typically take and how is it faster in this particular case?

8. In Results and Discussion' section "IRFinder-S integrates long read detection of IR", the sentence "In this long-read mode, we make multiple adjustments to the algorithm to account for the specificities of long-read data but also to account for the fact that these reads will often serve as the validation of IR and thus the default parameters are more stringent" does not clarify what sort of validation is associated with the long-read mode.

9- The explanation of the adaptation of the IRratio calculation to long-read sequencing is confusing. Although the authors refer that only "reads that fully cover the intron" are considered (Results), they refer to the usage of the "minimum coverage in the intron instead of the median" in the Methods. This, together with the aforementioned lack of detail of Figure 2B's caption (where it is not clear 1) what the red crosses represent or contribute to filter out and 2) how many intronic reads are considered as intronic abundance) hampers the understanding of the concept.

10. Related with the previous point and because the "main changes in the pipeline between short and long reads" is a critical message of the manuscript, Figure 2B should show the read count numbers corresponding to Ai and Ae.

11. It must be explained why three flanking positions for the vicinity of the exact annotated split nucleotides are used when quantifying IRratios for long reads. Is this (or can this become) a parameter set by the user?

12. In the sentence "Finally, the performance of our CNN may be underestimated because the misclassified IR events show mostly borderline and mislabeled introns (…)", "borderline introns" must be defined.

13. The use of boxplots is statistically meaningless for small sample sizes. The actual individual values must be plotted in Figure 4B.

14. In Results and Discussion' section "Implementation and validation of differential IR analysis", when describing results in Figure 4 on the comparison between differential intron retention found by DESeq2 and SUPPA2 and by qPCR, the authors must avoid confirmation biases by reinforcing "expected tendencies" if these results lack statistical significance ("With the exception of the intron in NR4A1, all the other events showed the expected tendencies, though not always with significant t-test p-values.").

15. Especially given the aforementioned lack of statistical power associated with the small sample sizes in Figure 4B, this should be complemented with a scatter plot relating the IRratios of IRFinder and qPCR for the 12 introns in the two conditions (e.g., keeping the two colours and using mean or median values with range whiskers). This would allow for a more robust assessment of the quality of validation.

16. In Figure 4C, there seems to be an enrichment of green points in the low IR ratio (bottom left) region. This observation must be explored and discussed in terms of specific biases of SUPPA2 or DESeq2 in dealing with IR events.

17. One of the presented main advantages of IRFinder-S is the usage of a "CNN filter to drastically reduce the false-positive rate of IR detection". This would be much clearer for the readers if the manuscript described, as an example, the biological context of an IR event that would be selected (particularly using short reads) if this CNN filter is not considered but is filtered out if it is. Although this edit will not change the main message of the manuscript, it will definitely contribute to improve its conveyance.

18. In Methods section "Convolutional Neural Networks", the sentence "we labelled as True Positive IR the introns with no warning, minimum coverage of 25 and IRratio above 0.1; as False Positive IR the introns with 50 minimum coverage and IRratio of 0" should be supported by the rationale of requiring double coverage for false positive events, especially in light of the scarcity of false positives noted just after.

19. We suggest that the solid fill of the histograms in Figure S1B is replaced by transparency or any solution that allows the four distributions to be intelligible and not to be perceived as cumulative.

Proofreading edits:

20. In Methods section "Convolutional Neural Networks" from Methods, "SR" is to be replaced with "short read" and "LR" with "long read". Similarly, in section "Differential Intron Retention", "CLI" is to be replaced by "command-line interface". The acronyms are not introduced neither they are standard in the field.

21. Please replace "mesenchimal" by "mesenchymal" in figure 4C.

**Reviewer 2**

**Were you able to assess all statistics in the manuscript, including the appropriateness of statistical tests used?**

Yes, and I have assessed the statistics in my report.

**Were you able to directly test the methods?**

No

**Comments to author:**

This manuscript describes an update on the IRFinder software published a few years in Genome Biology. This is probably the most popular software to analyze intron retention (over 100 citations), a regulatory process studied by more researchers every year. The additions to the software are good, and thus I would recommend publication. I have a few questions/suggestions, however:

1) If I understand well, IRbase only accepts hg38 analyses. Is that correct? If not, please clarify it in the text. If so, I strongly recommend making it available for other major species before publication.

2) Please clarify in the Results section that the DESeq2-based option of diff does not work with intron retention ratios, but with densities, whereas the SUPPA2-based option uses ratios. As it is written, it seems the DESeq2 option also models ratios, which would be incorrect.

3) Fig 4: While the validation of the trends of IRratios is good, the comparison between actual IRratios with IRFinder and qPCRs are quite poor in most cases, normally with higher IRratios calculated by IRFinder. Why would this be? Can this be benchmark with other available software to see if they all perform similar quantifications based on RNA-seq or is it an issue with IRFinder?

**Authors Response**

**Point-by-point responses to the reviewers’ comments:**

*It is with pleasure that we present a revised version of our manuscript: IRFinder-S: a comprehensive suite to discover and explore intron retention. We have addressed all the reviewers' comments point by point and we wish to thank them for their suggested experiments and comments on the text and figures which have resulted in a clearer and more concise manuscript. The main additions include making the code available on Code Ocean, an extensive benchmark against other algorithms with the addition of an independent experimental dataset, analysis of the utility of our CNN approach and enabling the analysis of non-human species through our software.*

Reviewer #1: The authors present a new suite of tools, IRFinder-S, for the detection and exploration of intron retention (IR) events from both short- and long-read sequencing of multiple samples, as well as for the detection of differential levels of IR between groups of samples. IRFinder-S builds on their previous software IRFinder and its highlighted novel feature is a convolutional neural network to reproduce expert identification of true IR events.

This manuscript is conceptually sound and IRFinder-S has the merit of implementing solutions for sharing and visualisation of data, leveraging the benefit of using long-read sequencing in the detection of IR events, and exploring the potential of deep learning in systematically reproducing expert examination of results. However, although we find no reasons to doubt that IRFindeR-S can satisfactorily (by current standards) quantify IR events, there are some shortcomings in the suite's benchmarking, detailed below, that need to be addressed for the edge brought by IRFindeR-S on the current state-of-the-art to be convincingly demonstrated. Moreover, we identified several needs of clarification, also listed below, that we encourage the authors to address.

Major concerns:

1. The deep learning model implemented to predict true, "actionable" IR events validated in a very specific biological context, as the training and testing steps were performed considering the EMT in the same cell line (MCF10A). Although there certainly are changes in gene expression and IR in that transition, the model is trained and tested on a limited set of events and therefore its evaluation is performed in a specific biological setting and not compared with any other scenario. For instance, would the predictive power of the model be similar if a different cell line was considered for external validation?

*We agree that, although many changes occur during EMT, our tests were run on a limited set of events. We included a second validation set on a different cell line, GM12878, a lymphoblastoid cell line. Again, we find that IRFinder-S, especially augmented by the CNN shows better results than our previous version and overall better results than the other benchmarked software.*

2. Along the same lines, the CNN filter to reduce false-positive intron retention events is applied only on IRFinder results. How does the described performance compare to that of applying the CNN to the calls of other computational tools available to perform IR detection?

*Applying the CNN filters to other software, namely iREAD, MAJIQ and Whippet, reduces the number of false positives (Suppl Fig S3). Even though IRFinder-S still comes out globally on top, adding the CNN to other algorithms can enhance other software.*

Other essential revisions:

3. The figure legends and captions need serious improvements, as they lack important clarifying details. Regarding Figure 1A, in the main text the authors propose "two types of tracks to visualize the IR events: a bar mode, showing the ratio values like a BedGraph and an IRFinder track to visualize the abundances of the flanking regions, the number of reads spliced and intron read depth" but the different visualization modes are not made explicit in its legend or caption.

*We include now a supplementary figure ( S1 ) showing the same locus of the same tracks with the BedGraph track view.*

4. Similarly, from the caption of Figure 2B alone, the reader is not able to grasp what sort of "sequencing errors" are depicted. There is a zoom-in effect represented but left undetailed on what it represents.

*We agree, the text now reads:*

*“Example of poor alignment to exonic borders due to sequencing errors that create jitter around the splice junctions.”.*

*We furthermore replaced the example with a clearer one, where the advantage of using the jitter function with error prone reads is more evident.*

5. In Figure 2A, the meaning and interpretation of the red crosses and the green lines should be made explicit in the caption or the figure itself.

*Agreed, the text now reads:*

*“Red crosses indicate regions that will be excluded from further analysis either due to other overlapping transcripts (left cross) or low mappability regions (right cross). Green lines indicate intronic abundance (Ai).”*

6. In Figure 2C, the caption sentence "due to the absence of reads covering the green box" is misleading, as long read mode will require reads that span across the full intron, i.e., both red and green boxes. The current version suggests that reads covering only the green box are be considered.

*The text now reads:*

*“due to the absence of reads covering the green and red boxes”*

7. In Results and Discussion' section "IRBase enables the visualization and contrast of IR events as well as data sharing", when referring to the improved upload process provided in the presented database, it is not clear what "fast labelling of experiments" actually means, making it difficult to assess how faster this is compared to other approaches. How long does this task typically take and how is it faster in this particular case?

*This was indeed misleading, we meant that the labelling was easy to execute by the user and flexible as they are added as free form text that can be subsequently used to manage sample groups. The text now reads:*

*“flexible labelling of experiments using free-form tags”*

8. In Results and Discussion' section "IRFinder-S integrates long read detection of IR", the sentence "In this long-read mode, we make multiple adjustments to the algorithm to account for the specificities of long-read data but also to account for the fact that these reads will often serve as the validation of IR and thus the default parameters are more stringent" does not clarify what sort of validation is associated with the long-read mode.

*We meant that long reads are often used in experiments as ground truth for the existence of IR events due to the fact that they span entire introns and their flanking exons. The probability of this occurring through sequencing or mapping error is near impossible.*

9- The explanation of the adaptation of the IRratio calculation to long-read sequencing is confusing. Although the authors refer that only "reads that fully cover the intron" are considered (Results), they refer to the usage of the "minimum coverage in the intron instead of the median" in the Methods. This, together with the aforementioned lack of detail of Figure 2B's caption (where it is not clear 1) what the red crosses represent or contribute to filter out and 2) how many intronic reads are considered as intronic abundance) hampers the understanding of the concept.

*The sentence was actually incorrect. The text now reads:*

*“Thirdly, we only consider the minimum read depth rather than the median when considering retained intron abundance.”*

10. Related with the previous point and because the "main changes in the pipeline between short and long reads" is a critical message of the manuscript, Figure 2B should show the read count numbers corresponding to Ai and Ae.

*Yes, we agree. We now include in the caption all the IRFinder measurements with and without the jitter function to clarify the advantage in case of error prone reads. The text now reads:*

*“Example of poor alignment to exonic borders due to sequencing errors that create jitter around the splice junctions. Without jitter (option -j 0 ), the software identifies only 110 SpliceLeft reads, 0 SpliceRight and 0 ExactSplice. With the jitter option ( -j 3), It identifies 183 SpliceLeft reads, 210 SpliceRight reads and 69 ExactSplice. Having an intron abundance (Ai) of 4 and using the ExactSplice as Ae, the IRratio in the first case is 1 and it raise the LowCover warning, in the second case the IRratio is 0.055, raising the MinorIsoform warning due to the imbalancement between the ExactSplice and the max(SpliceLeft, SpliceRight)”*

11. It must be explained why three flanking positions for the vicinity of the exact annotated split nucleotides are used when quantifying IRratios for long reads. Is this (or can this become) a parameter set by the user?

*We used three because of the codon size, but it can be set as a parameter.*

12. In the sentence "Finally, the performance of our CNN may be underestimated because the misclassified IR events show mostly borderline and mislabeled introns (…)", "borderline introns" must be defined.

*Agreed, the text now specifies:*

*“Finally, the performance of our CNN may be underestimated because the misclassified IR events are generally borderline with IRratios close to the threshold of 0.1, and mislabeled introns, due to incongruences between long and short read resolution power (Fig. S1B). “*

13. The use of boxplots is statistically meaningless for small sample sizes. The actual individual values must be plotted in Figure 4B.

*We included a scatterplot alongside the boxplot.*

14. In Results and Discussion' section "Implementation and validation of differential IR analysis", when describing results in Figure 4 on the comparison between differential intron retention found by DESeq2 and SUPPA2 and by qPCR, the authors must avoid confirmation biases by reinforcing "expected tendencies" if these results lack statistical significance ("With the exception of the intron in NR4A1, all the other events showed the expected tendencies, though not always with significant t-test p-values.").

*Agreed, the text now simply states:*

*“Of the 12 tested introns, 7 were confirmed using RT-qPCR.”*

15. Especially given the aforementioned lack of statistical power associated with the small sample sizes in Figure 4B, this should be complemented with a scatter plot relating the IRratios of IRFinder and qPCR for the 12 introns in the two conditions (e.g., keeping the two colours and using mean or median values with range whiskers). This would allow for a more robust assessment of the quality of validation.

*We included a scatterplot alongside the boxplot.*

16. In Figure 4C, there seems to be an enrichment of green points in the low IR ratio (bottom left) region. This observation must be explored and discussed in terms of specific biases of SUPPA2 or DESeq2 in dealing with IR events.

*We have added the following explanation in the text:*

*“This may be explained by the fact that events with low intronic coverage produce highly variable IR ratio values and may thus be harder to test for SUPPA.“*

17. One of the presented main advantages of IRFinder-S is the usage of a "CNN filter to drastically reduce the false-positive rate of IR detection". This would be much clearer for the readers if the manuscript described, as an example, the biological context of an IR event that filtered out if it is. Although this edit will not change the main message of the manuscript, it would be selected (particularly using short reads) if this CNN filter is not considered but is will definitely contribute to improve its conveyance.

*Indeed, we have now included an example where the CNN filters out a false positive IR result in figure S3*

18. In Methods section "Convolutional Neural Networks", the sentence "we labelled as True Positive IR the introns with no warning, minimum coverage of 25 and IRratio above 0.1; as False Positive IR the introns with 50 minimum coverage and IRratio of 0" should be supported by the rationale of requiring double coverage for false positive events, especially in light of the scarcity of false positives noted just after.

*We reasoned that it is easier to assert the existence of IR events than to assert their absence, thus we pushed the required coverage for negative events to 50 to increase their likelihood of being true negatives. This is now explained in the main text.*

19. We suggest that the solid fill of the histograms in Figure S1B is replaced by transparency or any solution that allows the four distributions to be intelligible and not to be perceived as cumulative.

*The histogram in Figure S1B was in a stacked form. We replaced it with four separate graphs, one for each class of result.*

Proofreading edits:

20. In Methods section "Convolutional Neural Networks" from Methods, "SR" is to be replaced with "short read" and "LR" with "long read". Similarly, in section "Differential Intron Retention", "CLI" is to be replaced by "command-line interface". The acronyms are not introduced neither they are standard in the field.

*Done*

21. Please replace "mesenchimal" by "mesenchymal" in figure 4C.

*Done*

Reviewer #2: This manuscript describes an update on the IRFinder software published a few years in Genome Biology. This is probably the most popular software to analyze intron retention (over 100 citations), a regulatory process studied by more researchers every year. The additions to the software are good, and thus I would recommend publication. I have a few questions/suggestions, however:

1) If I understand well, IRbase only accepts hg38 analyses. Is that correct? If not, please clarify it in the text. If so, I strongly recommend making it available for other major species before publication.

*Yes, that was the case. We have now made it possible for users to upload , analyze and visualize other species data but they cannot store them permanently in the database.*

2) Please clarify in the Results section that the DESeq2-based option of diff does not work with intron retention ratios, but with densities, whereas the SUPPA2-based option uses ratios. As it is written, it seems the DESeq2 option also models ratios, which would be incorrect.

*We clarified the concept in the results paragraph:*

*“[...]that allows end to end analysis using either the density based approach, DESeq2 [21], or the PSI based approach, SUPPA2 [22] adapted for IR ratios (material and methods)”*

3) Fig 4: While the validation of the trends of IRratios is good, the comparison between actual IRratios with IRFinder and qPCRs are quite poor in most cases, normally with higher IRratios calculated by IRFinder. Why would this be? Can this be benchmark with other available software to see if they all perform similar quantifications based on RNA-seq or is it an issue with IRFinder?

*IR ratios derived from RNAseq consider the median depth of the intron, meanwhile the ratios computed using qPCR consider only the depth in a specific position, that could cause a higher variability in the qPCR results. To verify this hypothesis, we compared the PSI values adjusted for IR computed using MAJIQ and, for the 9 out of 12 introns where the retention was identified, those values diverge similarly, from the qPCR results.*

**Second round of review**

**Reviewer 1**

We praise the authors for having addressed all our major concerns and responded to the minor issues we had raised. We still suggest some minor revisions that, in our opinion, will improve the manuscript’s intelligibility and get it effectively ready for publication.

1. We praise the authors for adding a new validation set. However, it is not made explicit which numbers, figure or table support the results mentioned in the main text (sensitivity of 0.81, specificity of 0.83 and balanced accuracy of 0.82). Information similar to that in Figure 3B, for the first set, should be provided.

2. The language used in the caption of Figure 2B still does not convey the relationship between long read-specific sequencing errors and the “jitter” effect. The sentence in the main text, which introduces jitter as a “slight imprecision in the definition of exonic boundaries” conveys the message more precisely. We suggest using, for the caption, something like “(…) due to sequencing errors that create a jitter effect on the mapping of long reads around the splice junctions”.

3. In Results and Discussion's section "IRBase enables the visualization and contrast of

IR events as well as data sharing", when referring to the improved upload process

provided in the presented database, we suggest that something like “user-defined/editable tags” replaces “free-form tags”, to stress that these are tags that can be easily changed by users.

4. The link made by the authors in responding to our doubt about the validation associated with the long-read mode should be included in the main text: long reads are often used for validation of IR events “because they span entire introns and their flanking exons”.

5. The following sentence, that the authors suggest in the rebuttal has been included in the caption of Figure 2B, is missing: “Having an intron abundance (Ai) of 4 and using the ExactSplice as Ae, the IRratio in the first case is 1 and it raise the LowCover warning, in the second case the IRratio is 0.055, raising the MinorIsoform warning due to the imbalancement between the ExactSplice and the max(SpliceLeft, SpliceRight)”. In this sentence, “imbalancement” can be replaced by “imbalance”.

6. Although the legend of figure 2B shows that one can set parameter -j as 0 and 3, this parameter is not mentioned in the text as one that can be tuned by the user. We suggest that this information is added together with the mention to “up to three nucleotide jitter in exonic boundaries (…)” being allowed.

7. Although Figure 4B has been improved by the dots next to the boxplots, our suggestion was to include one scatterplot showing the mean/median PIR values from IRFinder vs. the mean/median PIR values from qPCR, with the 12 cases (2 points – Epithelial and Mesenchimal – each) in the same plot.

8. It should be clarified why is it harder for SUPPA to test highly variable IR ratios with particularly low coverage.

9. To put execution times into perspective, it would be useful to compare them with the taken to align the corresponding FASTQ file, i.e., to go from FASTQ to BAM.

10. After assessing the code available from the Code Ocean platform, the difference between IRFinder and IRFinder-S should be made explicit in the README.md file. Assuming that IRFinder-S corresponds to the most recent version of IRFinder, we congratulate the authors for a very complete repository, with a Docker/Singularity image to facilitate running the program, a CHANGELOG file with the most recent updates and a thorough instructions' manual. However, there is no mention to this last version being “IRFinder-S”.

11. The acronym SR is never introduced in the article.

12. Caption of Figure 4C: this panel is not a dot plot, it is a scatter plot.

**Reviewer 2**

All comments have been properly addressed.

**Authors Response**

**Point-by-point responses to the reviewers’ comments:**

Reviewer #1: We praise the authors for having addressed all our major concerns and responded to the minor issues we had raised. We still suggest some minor revisions that, in our opinion, will improve the manuscript's intelligibility and get it effectively ready for publication.

1. We praise the authors for adding a new validation set. However, it is not made explicit which numbers, figure or table support the results mentioned in the main text (sensitivity of 0.81, specificity of 0.83 and balanced accuracy of 0.82). Information similar to that in Figure 3B, for the first set, should be provided.

*Done*

2. The language used in the caption of Figure 2B still does not convey the relationship between long read-specific sequencing errors and the "jitter" effect. The sentence in the main text, which introduces jitter as a "slight imprecision in the definition of exonic boundaries" conveys the message more precisely. We suggest using, for the caption, something like "(…) due to sequencing errors that create a jitter effect on the mapping of long reads around the splice junctions".

*Done*

3. In Results and Discussion's section "IRBase enables the visualization and contrast of

IR events as well as data sharing", when referring to the improved upload process

provided in the presented database, we suggest that something like "user-defined/editable tags" replaces "free-form tags", to stress that these are tags that can be easily changed by users.

*Users can add new tags but not change them: they are shared so modifying them would influence other user’s tags.*

*Replaced with “user-defined”*

4. The link made by the authors in responding to our doubt about the validation associated with the long-read mode should be included in the main text: long reads are often used for validation of IR events "because they span entire introns and their flanking exons".

*Done*

5. The following sentence, that the authors suggest in the rebuttal has been included in the caption of Figure 2B, is missing: "Having an intron abundance (Ai) of 4 and using the ExactSplice as Ae, the IRratio in the first case is 1 and it raise the LowCover warning, in the second case the IRratio is 0.055, raising the MinorIsoform warning due to the imbalancement between the ExactSplice and the max(SpliceLeft, SpliceRight)". In this sentence, "imbalancement" can be replaced by "imbalance".

*Done*

6. Although the legend of figure 2B shows that one can set parameter -j as 0 and 3, this parameter is not mentioned in the text as one that can be tuned by the user. We suggest that this information is added together with the mention to "up to three nucleotide jitter in exonic boundaries (…)" being allowed.

*Modified to:*

*“[...] we allow by default up to three nucleotide jitter in exonic boundaries when calculating correctly spliced introns (parameter -j). “*

7. Although Figure 4B has been improved by the dots next to the boxplots, our suggestion was to include one scatterplot showing the mean/median PIR values from IRFinder vs. the mean/median PIR values from qPCR, with the 12 cases (2 points - Epithelial and Mesenchimal - each) in the same plot.

*We tried to come up with a representation of one scatterplot with all 12 cases but this was hard to interpret. We have decided to stick to our current format.*

8. It should be clarified why is it harder for SUPPA to test highly variable IR ratios with particularly low coverage.

*Done.*

*“This may be explained by the fact that events with low intronic coverage produce highly variable IR ratio values. As a consequence the ratio values may be highly variable within replicates and methods such as SUPPA2 which make use of replicate variability to determine uncertainty may not produce statistically significant scores” .*

9. To put execution times into perspective, it would be useful to compare them with the taken to align the corresponding FASTQ file, i.e., to go from FASTQ to BAM.

*Done:*

*[...]however Whippet starts from FASTQ files instead of already aligned BAM files, whose alignment takes 120 minutes using STAR on a single core.*

10. After assessing the code available from the Code Ocean platform, the difference between IRFinder and IRFinder-S should be made explicit in the README.md file. Assuming that IRFinder-S corresponds to the most recent version of IRFinder, we congratulate the authors for a very complete repository, with a Docker/Singularity image to facilitate running the program, a CHANGELOG file with the most recent updates and a thorough instructions' manual. However, there is no mention to this last version being "IRFinder-S".

*Thanks :)*

*README on the github updated.*

*Once the Code Ocean capsule is published, we cannot modify it anymore the original one, but we sent a commit with a modified README file.*

11. The acronym SR is never introduced in the article.

Introduced at the first occurrence

“Because there was no short read (SR) dataset…”

12. Caption of Figure 4C: this panel is not a dot plot, it is a scatter plot.

*Corrected*
